# Handheld computers and the 21^st ^century surgical team: a pilot study

**DOI:** 10.1186/1472-6947-5-28

**Published:** 2005-08-18

**Authors:** Omer Aziz, Sukhmeet S Panesar, Gopalakrishnan Netuveli, Paraskevas Paraskeva, Aziz Sheikh, Ara Darzi

**Affiliations:** 1 Department of Surgical Oncology & Technology, Imperial College London, UK; 2 Division of Primary Care & Population Health Sciences, Imperial College London, UK; 3 Division of Community Health Sciences: GP Section, University of Edinburgh, UK

## Abstract

**Background:**

The commercial development and expansion of mobile phone networks has led to the creation of devices combining mobile phones and personal digital assistants, which could prove invaluable in a clinical setting. This pilot study aimed to look at how one such device compared with the current pager system in facilitating inter-professional communication in a hospital clinical team.

**Methods:**

The study looked at a heterogeneous team of doctors (n = 9) working in a busy surgical setting at St. Mary's Hospital in London and compared the use of a personal digital assistant with mobile phone and web-browsing facilities to the existing pager system. The primary feature of this device being compared to the conventional pager was its use as a mobile phone, but other features evaluated included the ability to access the internet, and reference data on the device. A crossover study was carried out for 6 weeks in 2004, with the team having access to the personal digital assistant every alternate week. The primary outcome measure for assessing efficiency of communication was the length of time it took for clinicians to respond to a call. We also sought to assess the ease of adoption of new technology by evaluating the perceptions of the team (n = 9) to personal digital assistants, by administering a questionnaire.

**Results:**

Doctors equipped with a personal digital assistant rather than a pager, responded more quickly to a call and had a lower of failure to respond rate (RR: 0.44; 95%CI 0.20–0.93). Clinicians also found this technology easy to adopt as seen by a significant reduction in perceptions of nervousness to the technology over the six-week study period (mean (SD) week 1: 4.10 (SD 1.69) *vs. *mean (SD) week 6: 2.20 (1.99); p = 0.04).

**Conclusion:**

The results of this pilot study show the possible effects of replacing the current hospital pager with a newer, more technologically advanced device, and suggest that a combined personal digital assistant and mobile phone device may improve communication between doctors. In the light of these encouraging preliminary findings, we propose a large-scale clinical trial of the use of these devices in facilitating inter-professional communication in a hospital setting.

## Background

Worldwide, there has been increasing interest in the use of wireless handheld technologies such as the personal digital assistant (PDA) in hospitals, with recent reports suggesting these devices may soon earn their position on a physician's desk next to the stethoscope [1]. A key reason for this interest is the possibility that PDAs can help facilitate safer decision making and improved efficiency of healthcare delivery [2].

The commercial development and expansion of mobile phone networks has led to the creation of many combined mobile phone and PDA devices, which could prove invaluable in a clinical setting. This is because these devices have the advantage of providing mobile information access, making it possible to retrieve clinically important information at any time of day and in any location. Information resources that doctors may benefit from include easy access to hospital addresses, protocols, evidence-based guidelines, textbooks, electronic patient records and drug formularies, to mention but a few [3,4].

Since being first introduced in 1948, the United Kingdom National Health Service (NHS) faces increasing costs as it attempts to provide free healthcare to all citizens of the United Kingdom, based on need rather than the ability to pay. Investment in information technology (IT) and communication infrastructure is an important part of healthcare expenditure as hospitals in the NHS aim to provide efficient and standardised healthcare delivery to a large patient population [5]. Nevertheless, a recent report by a parliamentary advice committee recommended that the NHS must overcome its preference for short term savings and develop strategies to stop the current underuse of new medical technologies. Currently, the UK only spends 0.36% of its gross domestic product (GDP) on medical technology, unlike Europe which spends 0.55% [6]. Hospital communication in the UK currently consists of pagers and landline telephones, and although they have had a ban on mobile phone use on their premises since the early 1990s (prompted by a warning issued by the UK Medical Devices Agency), many doctors ignore this, using their personal mobile phones for convenience at work.

With a greater appreciation that mobile phone and other wireless technologies are on the whole safe to use in the proximity of medical equipment [7], it is conceivable that devices combining the PDA and mobile phone could soon become the standard means for communication between hospital practitioners. The controlled use of mobile phone systems for hospital-specific communication by medical staff even in sensitive areas has already been deployed in a number of U.S. hospitals [8]. That said there is very limited and empirical evidence that investment in such technologies will actually translate into any clinically meaningful outcome. This pilot study aims to test the hypothesis that a PDA with a built-in mobile telephone is more efficient in facilitating communication between healthcare providers than a hospital pager device.

## Methods

The study group consisted of a heterogeneous team of doctors (n = 9) working in a busy surgical setting at the Academic Surgical Unit at St. Mary's Hospital (London). This unit was selected because it was a general surgical team with all members having a clinical commitment. There were varied levels of enthusiasm about the PDA, with some clinicians more critical and others more enthusiastic. All members of the team had a similar basic knowledge of computers and mobile telephones.

All members of the team were given a Palm Tungsten W™ PDA with mobile phone and web-browsing facilities, connected to a Vodafone UK network. In is important to note that these palm devices are no longer on the market and have been replaced by Treo™ smartphones. The Palm Tungsten W™ measures 12.1 × 7.79 × 1.65 cm, and weighs 184 grams, and has a 320 × 320 16-bit colour TFT display screen. The biggest connectivity feature of the Tungsten W™ (provided via a 1-cm long rounded Antenna) is the Class 10 GSM/GPRS radio [9].

This GSM/GPRS enabled PDA was connected to a Vodafone network. General Packet Radio Service (GPRS) keeps you permanently connected to the Internet but only charges you when your phone is sending or receiving data, and works at similar speeds to a dial-up modem on your home PC (100 kbps – 125 kbps) [10]. Global system for mobile communications (GSM) is an open, non-proprietary system that is constantly evolving. Voice is digitally encoded via a unique encoder, which emulates the characteristics of human speech. This method of transmission permits a very efficient data rate/information content ratio [11].

The Tungsten W™ is intended primarily as a data-centric device using the GPRS network. Any application that requires an Internet connection will automatically cause the device to establish a GPRS connection, which takes anywhere from 20–40 seconds depending on the signal strength, and the connection automatically shuts itself off after a period of inactivity. The device runs on a 33 MHz Dragonball VZ processor and includes 16 MB of RAM, of which 15 is available to the user. The manufacturer's specifications state that the lithium ion battery provides up to 10 hours of talk time [9].

All PDAs were also equipped with Dr. Companion^® ^software provided by MedHand^©^, containing electronic versions of commonly used UK medical reference textbooks such as the British National Formulary and the Oxford Handbook of Clinical Medicine. Other reference software available on this card included a drug interactions compendium, and anatomy atlas, the International Classification of Disease-10 guidelines, and medical calculators. Some users also chose to make use of the address book and diary functions of the PDA. However, detailed use of the various functions of the PDA was not monitored.

The crossover study was carried out for 6 weeks (17th May to 25^th ^June 2004), with the team having access to the PDA every alternate week. During weeks 1, 3 and 5, study participants adopted the conventional pager system for communication, and used PDAs during weeks 2, 4 and 6. The pagers used by the hospital were alpha-numeric with no internet text paging, and no two-way communication ability. The main feature of this device being compared to the conventional pager was its use as a mobile phone, but other features such as access to the internet and reference data were also considered and evaluated.

The primary outcome measure of interest for assessing efficiency of communication was the length of time it took for clinicians to respond to a call. To test this, one investigator, on a randomly selected day of the week, called all members of the team (n = 8 i.e. excluding the consultant) and measured the time taken for doctors to respond to the call. If after 5 minutes, there was no reply, it was noted that the doctor had 'failed to respond'. We used this measurement of 'response time' to measure efficiency of communication with the respective devices. The mobile phones were called directly and the paging was done via the standard paging system of using the hospital extension. In order to minimise the risk of bias we ensured that doctors could not differentiate a test call from regular communication traffic by making calls from different hospital extensions on each occasion. For the purpose of computing mean response time for each period of the study, the failure to respond was given a value of 301 seconds (5minutes + 1 second). Undoubtedly, not all members of the team would be in the same place; some were in the operating theatre, some on the wards and some in clinic. Whether this affected the response times is beyond the scope of this pilot-study. The aim was however to determine the response times at a randomly allocated point in time as in a real-life situation. We determined the rate ratios of those failing to respond for each pair of adjacent Pager/PDA periods and pooled the results using a fixed effects model to produce an overall rate ratio for failure to respond. This method was chosen so as to take into account the clustering of responses within individuals and the small sample size.

Furthermore, this study sought to assess the ease of adoption of new technology by evaluating the perceptions of the team including the consultant (n = 9), to PDAs by administering a questionnaire at the start of PDA use (week 2) and end of the study (week 6). The questions were adapted from the following validated rating scales: Computer Anxiety Rating Scale (CARS-C); the Computer Thoughts Survey Scale (CTS-C); and the General Attitude Towards Computer Scale (GATC-C) [12]. During the final week of the study, we also administered a questionnaire to the entire team which asked the user to evaluate the usefulness of the PDA and Dr. Companion^® ^software on a 5-point Likert scale (1 = not at all useful; 5 = extremely useful).

## Results

The response times to paging were measured on the eight participating junior doctors, although the number of participants was on occasions reduced because of night-duty and absence (Table 1). The point estimates of the response times were always smaller in the PDA weeks than in the pager weeks, but the wide confidence intervals made all but one comparison non-significant (significant difference in mean response times between pager week 5 and PDA week 4). However, when the rates of not responding to the bleep between adjacent pager/PDA periods were compared, in every comparison the periods of PDA use showed lower rates than those during the periods of pager use (overall RR: 0.44; 95%CI 0.20–0.93). We did not take into account the location of the respondents who may have been on the ward round or in the operating theatre, as we do not believe that this would alter the overall result.

Perceptions of nervousness associated with PDA use dropped significantly (p = 0.04) during the study, suggesting positive uptake of new technology by the team. Negative perceptions and disagreement (as measured by the validated questionnaires) regarding PDAs also decreased, albeit non- significantly [12]. Initially not all members of the team were keen to use the PDA-mobile phone device as they would have preferred using the traditional paging system owing to the reluctance to learn how to operate a new device. Dr. Companion^® ^software was rated as being moderately useful (mean = 3.77, SD 0.97) by the end of the study, with most doctors reporting using of the software between 5 to 10 times a day. Detailed usage of the various functions of the PDA and the software were not assessed, except that the favourite reference texts for most users were the British National Formulary, the Oxford Handbook of Clinical Medicine and the Evidence-based medicine (EBM) guidelines.

In addition, 7/9 (78%) of the staff preferred electronic-based reference material compared to paper-based material. 6/9 (67%) members of the team found the PDA to be user-friendly and easy to learn (67% and 56% respectively). 4/9 (44%) team members thought that it decreased their work load while 7/9 (78%) agreed that it enhanced the efficacy of communication between each other. However, 5/9 (56%) doctors did not think that the PDA gave them more time for other hospital tasks.

Finally, 7/9 (78%) agreed with the statement that having a PDA meant that they could deliver faster, more efficient patient care. Issues about battery life were raised by some members. Some participants charged their PDAs on a daily basis, others did not. Comments on battery life and poor signal strength in certain areas of the hospital such as the lifts were not explored in any further detail.

**Table 1:** Mean response time for each week of the study

Period	Device	Number		Response time (seconds)	
		Total^†^	Not responding	Mean	95%CI
1	Pager	8	4	182	70 to 293
2	PDA	7	2	97	0* to 266
3	Pager	6	2	133	0* to 277
4	PDA	7	0	18	4 to 33
5	Pager	6	3	178	34 to 322
6	PDA	7	2	92	0* to 224

^† ^Varies from week to week due to absence or night duty

* Adjusted to avoid implausible values

## Discussion

The use of combined PDA-mobile phone devices in our pilot study suggests that this technology might reduce the time doctors take to respond to a call. This is not surprising as the PDA with a mobile phone is a bi-directional device and enables faster communication between the caller and the doctor. Furthermore, it highlights an important potential limitation of pagers. Doctors often find themselves on busy hospital wards where phones are often otherwise engaged, or lifts, corridors, and just outside the hospital, where the absence of a landline makes the pager an ineffective communication device. Although the study lasted only six weeks, there is no evidence to suggest that these devices will not be as effective in the longer term. In addition, it is important to note that the members of the study group were not all 'PDA enthusiasts', but a rather diverse group consisting of pro-PDA users as well as sceptics even though it is often felt that resident physicians or surgeons are more likely to use newer technology and are more receptive to change. The group on the whole was largely heterogeneous in this respect. Only 2/9 (22%) participants in the study had prior experience with a PDA (1 using a Palm device and 1 using a Pocket PC device).

The assessment of doctors' perceptions regarding introduction of PDAs during the six-week study demonstrated an increasing level of confidence with the devices. This is an important characteristic that any device proposed to replace the hospital pager must have. Widespread mobile phone and PDA use in the commercial setting have meant that the majority of doctors working in the UK already own such technology and are therefore likely to be comfortable with using the devices at work.

The ability to store electronic versions of commonly used reference material such as the British National Formulary and the Oxford Handbook of Clinical Medicine on a single chip is a potentially valuable attribute of the PDA. It means that clinicians can now carry on their person a quantity of reference material that would previously have been inconceivable in hard-copy format. This is, we believe, particularly important for junior doctors and during night-time periods when there are fewer people around to ask for advice. With regards to standard of patient care, it is conceivable that these reference materials may aid correct diagnosis and management, particularly with regards to correct drug dosage.

A major limitation of most research involving mobile phone devices in healthcare is that this has been carried out under laboratory conditions, as opposed to a field test such as in this study [13]. Research on the use of PDAs however, has been in the form of field tests, and supports the finding of this study. An example of this is a recent trial in the United Kingdom, where authors looked at the use of PDAs to access reference information as well as local and national guidelines by members of an on call service for health protection, with results suggesting that this was a fast, reliable and easily maintained source of information that could be used by other groups of practitioners [14].

## Conclusion

PDAs have been increasingly used in clinical medicine for the delivery of information at the point of care, the collection of patient information, updating of clinical records, electronic prescribing and medical education. This pilot study integrated a combined PDA and mobile phone device into the daily schedule of a team of general surgeons, showing its usefulness primarily as a communication tool. Although we were unable measure a direct patient benefit from the use of these devices, the majority of doctors in the trial felt that having a PDA with a mobile phone as an in-built feature did help them to deliver better health care, and found this technology easy to adopt.

In light of these promising initial findings, we now propose a large-scale clinical trial of the use of PDAs with built-in mobile telephones in the hospital setting. This may be a first step in developing the evidence base for a new hospital communication system that may eventually replace the quaint, but hopelessly outdated hospital pager. Other wireless technologies such as blue-tooth and wireless local area networks must also be considered in any communication system proposed for use in hospitals.

## Competing interests

The author(s) declare that they have no competing interests.

## Authors' contributions

Omer Aziz concieved and coordinated the study. Sukhmeet S. Panesar was responsible for data acquisition, statistical analysis, and write-up. Gopalakrishnan Netuveli and Aziz Sheikh were responsible for study design and statistical analysis. Paraskevas Paraskeva and Ara Darzi were responsible for study implementation, result interpretation, and oversaw the trial.

## Pre-publication history

The pre-publication history for this paper can be accessed here:


